# Are Dropped Bone Grafts Safe to be Re-used? - An Experimental Study Comparing Efficacy of Chlorhexidine, Povidone-Iodine and Alcohol

**DOI:** 10.5704/MOJ.2107.011

**Published:** 2021-07

**Authors:** MF Mat-Salleh, AN Sadagatullah, MY Ibrahim, I Abdul-Aziz, WA Wan-Abdullah, N Maning, MN Md-Hassan, MR Ab-Rashid

**Affiliations:** 1 Department of Orthopaedics, Universiti Sains Malaysia, Kubang Kerian, Malaysia; 2 Department of Orthopaedics, Hospital Raja Perempuan Zainab II, Kota Bharu, Malaysia; 3 Department of Pathology, Hospital Raja Perempuan Zainab II, Kota Bharu, Malaysia

**Keywords:** contaminated bones, chlorhexidine, disinfectant

## Abstract

**Introduction:**

A dilemma arises when a bone graft or fracture fragment is accidentally dropped on the operation theatre floor and becomes contaminated. This study aimed to determine the efficacy of simple and readily available antiseptic solutions in disinfecting contaminated bones.

**Material and Methods:**

This experimental study involved 225 bone specimens prepared from discarded bone fragments during a series of 45 knee and hip arthroplasty surgeries. The bone fragments were cut into five identical cubes and were randomly assigned to either control (positive or negative), or experimental groups (0.5% chlorhexidine, 10% povidone-iodine or 70% alcohol). The control negative was to determine pre-contamination culture. All bone specimens, except the control negative group were uniformly contaminated by dropping on the operation theatre floor. Subsequently, the dropped bone specimens except for the control positive group, were disinfected by immersing in a respective antiseptic solution for 10 minutes, before transported to the microbiology laboratory for incubation.

**Results:**

The incidence of a positive culture from a dropped bone fragment was 86.5%. From the 37 specimens sent for each group, the incidence of positive culture was 5.4% (2 specimens) after being disinfected using chlorhexidine, 67.6% (25 specimens) using povidone-iodine and 81.1% (30 specimens) using alcohol. Simple logistic regression analysis demonstrated that chlorhexidine was significantly effective in disinfecting contaminated bones (p-value <0.001, odd ratio 0.009). Povidone-iodine and alcohol were not statistically significant (p-value 0.059 and 0.53, respectively). Organisms identified were Bacillus species and coagulase negative Staphylococcus. No gram-negative bacteria were isolated.

**Conclusion:**

A total of 0.5% chlorhexidine is effective and superior in disinfecting contaminated bones.

## Introduction

A challenging situation arises when a bone graft or fracture fragment is inadvertently dropped onto the operation theatre floor and becomes contaminated. Graft contamination is common during surgery^1^. A total of 75% of graft contamination was due to a graft falling on the floor^2^. An orthopaedic surgeon facing this unexpected situation needs to make a judicious judgement whether to discard the dropped bone or to decontaminate and continue utilising it. Electing to dispose of the contaminated graft, then harvesting autologous bone graft or using synthetic or allograft implies cost and morbidity to the patient. Another group of surgeons would prefer to disinfect and continue using the dropped bone, after taking into consideration the safety in regard to the risk of infection associated with contaminated bones.

An ideal method of decontamination is applying sterilisation which destroys any organism and at the same time without imposing a harmful effect on the viability of osteoprogenitor cells. Furthermore, an ideal method of decontamination should also be safe, quick and will not interrupt the whole process of surgical procedure^3^. Using autoclave, gamma irradiation and ethylene oxide for sterilisation are not preferred as they are expensive, time consuming and have harmful effect on graft material^4-6^. A simpler, cheaper and readily available option in the operation theatre will be the disinfection using an antiseptic solution such as chlorhexidine, povidone-iodine or alcohol.

To be able to weigh the possible risk and benefit, a surgeon who decides to undertake disinfection of a dropped bone would need to know if it had become contaminated. It was believed that a dropped bone graft on the floor yield no positive culture^7^. However, Bruce *et al* reported 70% of contamination rates in dropped bones for 30 secs^8^. Yazdi *et al* demonstrated 44% incidence of positive cultures in contaminated bones^9^.

The next concern when considering to disinfect a contaminated bone is the effectiveness of the antiseptic solutions available in the operation theatre. Bauer *et al* experimented contaminated bone grafts and reported that chlorhexidine and dry povidone-iodine decontaminated all bone samples, but not wet povidone-iodine^10^. Yazdi *et al* claimed that chlorhexidine 0.4% was the best antiseptic solution for contaminated bones in rabbit^9^. Hooe *et al* found that 10% povidone-iodine to be superior to 4% chlorhexidine solution in decontaminating graft soiled with *Pseudomonas aeruginosa* and *Staphylococcus aureus*^11^. On the other hand, Goebel *et al* found that 10% povidone-iodine and triple antibiotic solutions were ineffective in decontaminating rabbit bone patellar tendon bone grafts^12^.

In our current local setting, when the surgeons encounter the incident of an unintentional drop of a bone graft during surgery, disinfection using povidone-iodine is the common practice. Centeno *et al* reported that 10% povidone-iodine solution was the most popular antiseptic solution used in disinfecting dropped grafts among a group of 223 surveyed surgeons^2^. This is an interesting finding given the fact that the current practice seems to contradict what the literature ascertains, which demonstrated that 4% chlorhexidine solutions to be the most effective methods of decontamination^12^. This data is also consistent with a later study by Bruce *et al*, reporting that 4% chlorhexidine as the most effective decontaminating agent^8^.

The general objectives of this study were to determine the efficacy of different antiseptics readily available in our local hospitals, specifically to compare the effectiveness of 0.5% chlorhexidine, 10% povidone-iodine and 70% alcohol in disinfecting contaminated bones. In addition, this study was designed to document the incidence of positive cultures from the dropped bone fragments and to identify common bacteria in contaminated bones. The findings from this study will help the clinician to develop a protocol that can be used as a guide in choosing the best antiseptic solution in disinfecting contaminated bones, should they encounter this challenging circumstance in their practice.

## Materials and Methods

A preliminary study was conducted prior to performing the experimental study. The rationale of the preliminary study was to evaluate the degree of contamination on the operation theatre floor, represented by the bacterial load in regards to the duration of surgery. The data will be analysed to determine the best time to drop the bones during the experimental study.

The preliminary study involved six sets of floor swabs during knee or hip arthroplasty surgery. One set consisted of four swabs taken were; (1) before the surgery started (after floor cleaning), (2) at 30 mins, (3) at 1 hour and (4) at 2 hours after the surgery has started^8^. Floor swabs were taken within the parameter of one-meter radius from the operation theatre table, using sterile cotton swab sticks, which were subsequently streaked on the agar plates, before being transported to the microbiology lab for incubation to determine number of bacterial colonies.

**Fig. 1:**
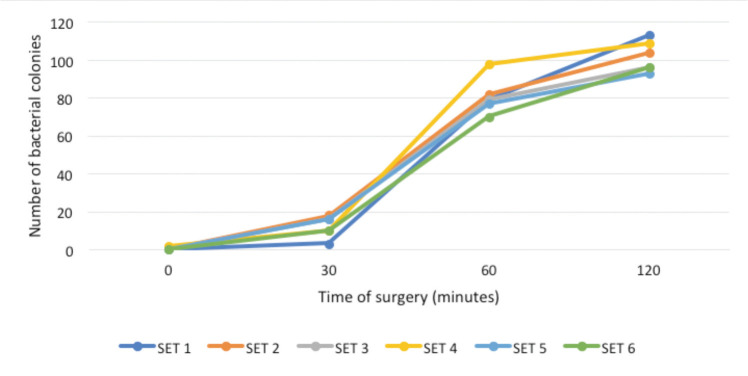
Graph showing total bacterial count (colonies) according to time of surgery.

All floor swabs taken before the operation started yielded no bacterial growth, except one swab that had two colonies of coagulase negative *Staphylococcus*. As the surgery progressed, the bacterial colonies on the blood agar increased. The bacterial load on the operation theatre floor amplified dramatically after 60 minutes of surgery had started (Fig. 1).

Data which were analysed via IBM SPSS statistic software [version 22] using simple linear regression analysis, demonstrated a statistically significant positive association between bacterial load on the operation theatre floor and duration of surgery (p<0.001) (Table I). It was decided that 60 minutes was the optimal time to drop bone specimens due to the high likelihoods of getting contaminated and taking into consideration the average duration of orthopaedic surgeries.

**Table I:** Simple linear regression analysis showing the relationship between the total bacterial count on the operation theatre floor and time of surgery

Variables	ß	95% CI	p-value	r^2^
Time of surgery (mins)	0.913	0.746, 1.080	0.0001	0.854

*Bacterial count on the operation theatre floor is significantly and positively associated with time of surgery (p<0.0001) and it also explains about 85.4% of the variability (r^2^=0.854). In our study sample, for each minute increase in time of surgery, on average, bacterial count increase by 0.913 colonies.

The experimental study involved discarded bones taken during knee or hip arthroplasty surgery for the treatment of osteoarthritis or femoral neck fractures. The arthroplasty surgeries were total knee and hip replacement, and hemiarthroplasty of the hip. Bone specimens from patients with a history of infection, bone tumour, connective tissue disease or patients undergoing revision arthroplasty surgery were excluded from the study. Bone specimens from control negative groups that had positive culture were also excluded from the study.

**Fig. 2:**
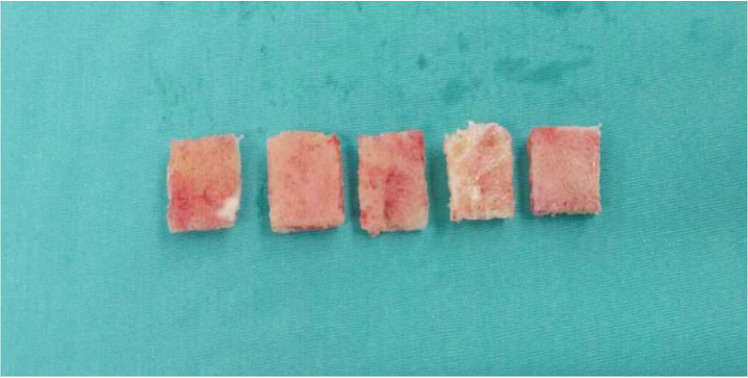
Bones specimens were prepared in uniform cubes size.

The study was conducted in a standardised operation theatre; environment where the standard operating procedure for arthroplasty surgery applied. The bone fragments underwent a sterile and uniform preparation. Soft tissue attachments were removed using a blade and rongeur. The cartilage was removed to leave only fragments of cancellous bone. Then, the cancellous bone fragments were cut into the same size of cubes (dimension 2.0cm X 1.5cm X 0.5cm) (Fig. 2), using either a bone cutter or mallet and osteotome. The bone specimens were cleansed from blood clots using sterile wet gauze and then wiped using sterile dry gauze.

The bone fragments were prepared in uniform size and shape and then were placed in identical containers prior to labelling for the various treatment prior to culture in no particular order. This ensured the specimens were randomly assigned one of five groups, two control groups (positive or negative) and three experimental groups (chlorhexidine, povidone-iodine or alcohol).

The control positive group were dropped and contaminated bone specimens. They were sent to the laboratory without undergoing a decontamination process. The control negative group were pre-contamination specimens to ensure strict sterility of all bone specimens. They were sent for microbiological studies without undergoing the process of contamination and disinfection. A total of 0.5% chlorhexidine in aqueous (1:200), 10% povidone-iodine and 70% alcohol were used as they are commonly available in the operation theatre in the local hospitals. All specimens except the control negative group were contaminated by dropping onto the floor, in the parameter of one-meter radius from the operation table. This area had the highest bacterial load and high risk of contamination as it was largely occupied by the doctors and staffs during surgery. The bone specimens were dropped on the floor at 60 minutes after surgery started, from the height of one meter, which is the average height of the operation table^9^. 60 minutes were determined from the data analysis of the preliminary study (Fig. 1, Table I). Each of the bone specimens was then collected using sterile forceps after 30 secs resting on the floor as advocated by Bruce *et al*^8^, which is the simulated time taken for a surgeon to decide, discover and collect the dropped bone specimens. All dropped specimens except the control positive group were disinfected by immersing in a respective antiseptic solution for 10 minutes and then rinsed with sterile saline for 15 seconds, as done by other researchers^9,13,14^. The specimens were immediately transported to the microbiology lab in a nutrient broth media for incubation.

We have calculated the required sample size by using a Power and Sample size program (PS 3.0) based on a power of 80%. Incidence of positive culture was set to be 0.7, based on data taken from a previous study^8^. Considering 10% drop out rate, our minimum required sample size was 170 bone specimens. As we have decided that one knee or hip procedure will contribute to one set of 5 bone specimens, our study will involve a minimum of 34 knees or hips procedures. The results from the experimental study were then analysed via IBM SPSS statistic software [version 22] using simple logistic regression analysis.

## Results

A total of 45 sets of bone specimens making up to a total of 225 sterile bone specimens were collected from 45 procedures involving knee and hip arthroplasty surgeries.

Each of the study group were allocated 45 bone specimens. Two specimens from the control negative group had a positive culture, hence the data from this set of bone specimens were excluded from the study analysis. Six sets of bone specimens had a drop out during the process of incubation (three were missing and three had equipment malfunction). Thirty-seven sets of bone specimens (total of 185 specimens) were incubated to determine bacterial growth and data were analysed according to the assigned group.

Thirty-two specimens from the control positive group had a positive culture. The incidence of a positive culture from dropped bone specimens was 86.5% [95% CI (75.5, 97.5)]. Two bone specimens from the chlorhexidine group had positive culture, with an incidence rate of 5.4% [95% CI (0, 12.7)]. Twenty-five specimens in the povidone-iodine had positive culture, incidence rate 67.6% [95% CI (52.5 – 82.7)]. Whereas, 30 specimens from the alcohol group had positive culture, making the incidence rate of 81.1% [95% CI (68.5 – 93.7)] (Table II).

**Table II:** Incidence rate of positive culture according to groups

Group	Growth	Incidence rate	95% CI
Control (positive)	32	86.5%	75.5, 97.5
Chlorhexidine	2	5.4%	0, 12.7
Povidone-iodine	25	67.6%	52.5, 82.7
Alcohol	30	81.1%	68.5, 93.7

Total bone specimens N=37 per group, CI=confidence interval

Simple logistic regression analysis demonstrated that chlorhexidine was significantly effective in disinfecting contaminated bones (p-value <0.001, odd ratio 0.009). Povidone-iodine and alcohol, on the other hand, were not statistically significant (p-value 0.059 and 0.53, respectively) (Table III).

**Table III:** Simple logistic regression analysis showing the effectiveness of antiseptics in experimental groups

Antiseptics	Crude OR (95% CI)	p-value
Chlorhexidine	0.09 (0.002, 0.49)	0.0001
Povidone-iodine	0.326 (0.101, 1.046)	0.059
Alcohol	0.670 (0.192, 2.34)	0.530

A total of 93% of the organism identified from the positive culture of the experimental groups were gram positive cocci while only 7% were gram positive bacilli. Gram positive bacilli and cocci were both present in the control positive group. There were no gram-negative bacteria isolated. Coagulase test performed demonstrated that all gram-positive cocci bacteria were coagulase negative *Staphylococcus. Bacillus* was grown from both specimens that had a positive culture in the chlorhexidine group. *Bacillus* species were cultured from 21 of 25 specimens with positive culture in the povidone-iodine group (84%), while four specimens were coagulase negative *Staphylococcus* (16%). Similar to the chlorhexidine group, 100% of positive cultures from the alcohol group were *Bacillus* species.

## Discussion

From our study, the risk of contamination of a dropped bone during surgery is 86.5%. Previous studies conducted in the 90’s investigated the risk of contamination in dropped bones and found a contradicting result, which was very low contamination rates, zero percent^7^ and 10%^15^. Nonetheless, very high contamination rates of 90% were observed in contaminated bone-tendon grafts^16^. Burd *et al* postulated the wide range of results were due to low infection rate occurred in contamination of bone alone whereas higher infection rates occurred in contamination of both bone and tendon^13^. A recent study examined dropped osteoarticular bone fragments and found a contamination rate of 70%^8^, which is in keeping with our findings.

The duration of the surgery when the graft is dropped is an important factor influencing the risk of contamination. Our preliminary study demonstrated that the bacterial load on the operation theatre floor amplified as the time of the surgery progressed. Most of the falls during surgery occur towards the end of a surgical procedure^17^. A bone is likely to be accidentally dropped later rather than earlier on during the surgery hence a higher risk of becoming contaminated. Due to the high risk of infection, surgeons need a correct and impromptu decision either to discard and use other bone grafts which would reflect extra resources or to decontaminate with an effective disinfectant. Choosing to decontaminate rather than to discard an indispensable graft such as large intraarticular fragments might be the only option a surgeon has.

Our study discovered that 0.5% chlorhexidine is effective in disinfecting contaminated bones (p<0.001). Chlorhexidine disinfected all bones except two specimens (incidence rate of 5.4%). Similar findings were testified by Yazdi *et al* using 0.4% chlorhexidine, although they reported that the difference was not statistically significant with povidone-iodine^9^. However, povidone-iodine and alcohol were shown to be not significant in our study. High incidence rate of positive culture was observed in these experimental groups. Out of 37 specimens, povidone-iodine failed to disinfect 25 while alcohol 30 specimens (incidence rate 67.6% and 81.1%, respectively).

The organism incubated from the positive culture were gram positive bacilli and cocci, whereas no gram-negative organism identified. All gram-positive cocci organism were coagulase negative *Staphylococcus*, which made up 93% of all organisms identified. These findings were similar with Bruce *et al*, reporting the figure to be 97%^8^. *Bacillus* species were the second common organism, which represented only 7%. On the contrary, Bruce *et al* identified a small number of gram-negative organisms (less than 1%)^8^. On the other hand, a study of bacteriology in the operation theatre was conducted and they found the presence of a high concentration of *Staphylococcus aureus*, in addition to coagulase negative *Staphylococcus* and *Bacillus* species^18^.

All contaminated specimens from the chlorhexidine and alcohol group grew *Bacillus* species. A total of 21 of 25 positive culture (84%) in the povidone-iodine group were *Bacillus* species. Out of 37 specimens, although chlorhexidine successfully disinfected 35 bone specimens (94.6%) while alcohol only disinfected seven specimens (18.9%), both antiseptics failed to decontaminate all *Bacillus* organism. Povidone-iodine failed to disinfect both organisms, with a large percentage of *Bacillus* (84%) and a smaller percentage of coagulase negative *Staphylococcus* (16%). The pattern of the contamination may suggest that *Bacillus* species is a highly resistant organism presented on the operation theatre floor. Our study proved that chlorhexidine is an effective disinfectant and its ability to decontaminate all coagulase negative *Staphylococcus*. Although chlorhexidine has great bactericidal properties, it is not effective against spores and mycobacterial species^19^. In our study, this is evident by its inability to decontaminate all *Bacillus* species, which is a spore-bearing bacteria.

We conducted the study in the operation theatre environment which is sterile and the bones specimens were dropped at 60 mins when the surgery is still ongoing. Being able to perform the experiment in a real situation is the strength of our study. In addition, by excluding positive culture specimens from the control negative groups , we ensured strict sterility of the bone specimens. It was a surprise when two specimens in our study from control negative groups grew organisms. The two patients from whom we took the specimens were retained in the ward postoperatively for a longer course of intravenous antibiotics.

We immersed the dropped bone in the respective antiseptic solution for 10 minutes. In other studies, Yazdi *et al* immersed the contaminated bones in 20 minutes^9^ and Bauer *et al* ensured two minutes of contact time with antiseptics^10^. Soyer *et al* decontaminated bones infected with *Staphylococcus epidermidis* for a different exposure time up to 10 minutes with povidone-iodine^14^. Our study demonstrated that immersing in 10 minutes chlorhexidine able to disinfect all but two contaminated specimens. We found that 10 minutes to be practical in the real situation should a surgeon decide to disinfect a contaminated bone graft, considering its possible interruption of the intended surgical procedure. Unfortunately, there was no study exploring the effect of different duration of exposure to chlorhexidine, hence the need for further studies.

Despite the superiority of chlorhexidine as a disinfectant, we did not explore its safety in regards to osteoprogenitor cell viability. Perhaps a surgeon considering to disinfect a contaminated bone would like to know the cytotoxic effect of different antiseptics on the grafts, in which unfortunately we were unable to carry out further examination such as histological studies, to document any structural changes within the sample cells. Nonetheless, it was reported that chlorhexidine gluconate has the potential effect on polymorphonuclear granulocyte toxicity and impaired phagocytic efficiency^20^, which could impair the healing process of bone. Chlorhexidine mainly affects cell growth while povidone-iodine affects matrix mineralisation of alveolar bone cells^21^. Furthermore, Bruce *et al* demonstrated cleansing with povidone-iodine to provide an optimal balance between effective decontamination and cellular toxicity, in which the greatest number of live cells was retained^8^.

Bauer *et al* reported that mechanical agitation in wet povidone-iodine was more superior to chlorhexidine in balancing the effectiveness of disinfection and maintenance of tissue viability^10^. Unfortunately, we did not explore other modalities such as mechanical agitation in our study. This study was reproduced among the reasons were to enrichen the method of the previous authors and re-evaluate the practices and available methods and also to reproduce the same or equal results in our operative theatre settings. The methods are experimental, and the results were true to the operative milieu of our centre. Nonetheless, further expansion of the study would be beneficial for the expansion of knowledge.

The bacterial count increases approximately 100-fold with heavy traffic in the room^22^. We did not further quantify the number of bacterial colonies presented in the positive cultures in the experimental groups. However, in our study, by following the standard operating procedure for arthroplasty surgery, the average number of personnel in the operation theatre during the surgery were kept at six to eight persons at a time, hence controlling the rate of contamination on the operation theatre floor.

Last but not least, the wide confidence interval for povidone-iodine and alcohol groups indicates that the sample size was not adequate for this study (Table III). The upper limit for the povidone-iodine is close to one (1.046) and the p-value is very close to 0.05 which means that they were likely to be effective too if the sample size was larger. We realised it as a shortcoming of the study, which can be improved in the future.

## Conclusion

Although the literature demonstrated that povidone-iodine was the most commonly used antiseptic in disinfecting dropped bone graft, the superior effectiveness of the 0.5% chlorhexidine in our study signifies a change in the current practice. Similar outcomes could be extrapolated in intra-operative decontamination of dropped instruments or implants using chlorhexidine. Further studies are required to assess the safety of chlorhexidine in regards to cell viability, which is a limitation of our study.
